# Urban South African Adolescents’ Perspectives on Healthy and Unhealthy Foods and the Drivers of Their Food Choices in Their School Food Environment: A Pilot Study

**DOI:** 10.3390/ijerph23020208

**Published:** 2026-02-07

**Authors:** Alice Scaria Khan, Francesca Dillman-Carpentier, Elizabeth Catherina Swart

**Affiliations:** 1 School of Public Health, University of Western Cape, Bellville, Cape Town 7535, South Africa; 2 Hussman School of Journalism and Media, University of North Carolina at Chapel Hill, Chapel Hill, NC 27599, USA; francesca@unc.edu; 3 Department of Dietetics and Nutrition, University of Western Cape, Bellville, Cape Town 7535, South Africa

**Keywords:** adolescents, school food environment, healthy and unhealthy foods, food choices, metropolitan and nonmetropolitan urban areas

## Abstract

Background: Childhood obesity is on the rise in South Africa and adolescents spend a substantial amount of time in the school food environment (SFE), which plays a role in shaping their food choices and provides a critical setting to improve diets. Objective: To investigate South African adolescent school-going learners’ knowledge and understanding of healthy and unhealthy foods and the drivers of their food choices in their (SFE). Design: Qualitative participatory research methods including workshops, photovoice and focus group discussions (FGDs). Setting: Two urban public high schools, one non-metropolitan and one metropolitan, in two separate provinces (Eastern Cape and Gauteng) in South Africa. Participants: Adolescents 14–18 years (n = 42). Results: Unhealthy ultra-processed foods (UPFs) were found to be rampant in the SFE, and healthy foods were scarce, limiting learners’ choices. Taste preference was a major driver of adolescent food choices as were satiety, value for money, affordability, convenience, visual appeal and seeming “cool or “rich” by purchasing branded franchise fast foods. Learners had some general nutrition knowledge, but this did not translate into healthy food choices. Banning unhealthy foods in the SFE and providing affordable and satiating healthy foods were proposed as solutions. Conclusions: UPFs such as packaged foods and fast food were considered tasty but unhealthy, yet were preferred. Interventions are needed to promote healthy diets by changing the SFE, and eventually adolescent food choices. This will require government regulation banning the sale of unhealthy food and beverages (F&Bs) in the SFE and subsidising healthy satiating foods to change dietary behaviour.

## 1. Introduction

Proper nutrition plays a vital role in supporting adolescents’ health, growth and development, and there have been growing global efforts to tackle all forms of malnutrition [1]. Overweight and obesity during childhood, including adolescence, can have a serious social and psychological impact due to the increased risk of depression, weight stigma, low self-esteem, peer bullying, eating disorders, poor school performance or anxiety [2,3,4,5]. A key determinant of obesity is diet, the interaction between human food preferences and the environment in which these preferences are learned and expressed [6].

Adolescence is a transitional phase marked by a shift from dependence on adults to the development of independent dietary habits [7]. To effectively promote healthy eating among adolescents, it is imperative to develop a comprehensive understanding of their dietary intake and food choice behaviours across diverse sociocultural and environmental contexts [7].

Contemporary urban food environments have been associated with the ongoing nutrition transition, marked by increased availability, accessibility and marketing of unhealthy ultra-processed foods (UPFs) high in fat, salt and sugar, particularly in sub-Saharan Africa [1,7]. The transition to unhealthy dietary patterns has been observed among adolescents living in urban low-income areas [8]. Urbanisation has changed dietary patterns from traditional, plant-based diets—rich in whole grains, legumes, fruits and vegetables—towards increased consumption of less healthy diets that are low in fibre, highly processed, and excessive in terms of sugar, fat, salt, energy and animal-derived proteins [9]. Furthermore, children and adolescents are more inclined to consume UPFs than older adults [10].

Childhood obesity is increasing in South Africa, and if the current trend continues, the country’s adolescents are projected to rank as the twelfth most obese worldwide by 2030 [11]. Adolescents, girls in particular, in South Africa are subject to a mounting obesity crisis [12]. The proliferation of multinational food companies in South Africa adds to the obesity crisis [13]. A study on adolescents’ perspective of drivers of obesity in the Western Cape province in SA found that accessibility to unhealthy foods and the advertising of these foods were factors in increasing their purchase and consumption [14].

A study in the Eastern Cape province of SA found that unhealthy energy-dense foods were the main items purchased around school, and that healthy options were rare [15,16]. This supports previous findings by Faber et al. that learners seldom pack lunchboxes with healthy foods and vendors and tuckshops in the SFE predominantly sell unhealthy snacks and drinks [17]. A study in the province of KwaZulu-Natal reported a lack of control over unhealthy foods being sold around schools [18]. The National School Nutrition Programme was found to provide meals to most learners, but the SFE was not supportive of healthy eating [15]. Poverty and high food prices have been reported as barriers to healthy food purchases in SA [17]. A review of 38 studies conducted on the SFE globally found a shared theme across a majority of the studies indicating the availability of cheap, convenient foods high in saturated fat, salt and sugar sold in school tuckshops, with considerable scope to improve the availability of healthy choices within SFEs [19]. Globally, the cost of healthy foods in SFEs was found to be greater than the cost of unhealthy foods [20,21,22].

Adolescents may perceive the factors influencing their dietary behaviours differently from adults, creating a pressing need for more evidence reflecting their perspectives. Globally, there are increasing calls to amplify adolescent voices and their active participation in actions to promote healthy dietary behaviours [7]. Children are exposed to food and beverage (F&B) marketing in the spaces where they live, learn and play [23,24,25], and the school food environment (SFE) plays a crucial role in adolescent food choices [26], especially considering the amount of time learners spend at school. In this context, it is critical that adolescents’ knowledge of healthy and unhealthy foods, and the drivers of their food choices and consumption, is explored through their lived realities.

Qualitative research on food environments remains underutilised, despite its significant potential, especially in under-researched contexts such as low- and middle- income countries (LMICs) [27]. Lived experiences of food environments may expose deeper insights into the factors that shape food purchases and consumption [27]. Photovoice is a visual methodology that uses photographic documentation of people’s everyday lives as an educational tool to record and reflect on their needs, encourage action and promote critical dialogue to reach policymakers [28]. It is a powerful medium for group engagement, knowledge sharing and advocacy for social reform [29]. Photographs are taken by participants within a specific setting (in this case the SFE) and used as a prompt for them to describe their lived realities [28]. In this study we aim to understand adolescent learners’ understanding of the healthfulness of commonly consumed foods in SA, their recognition and consumption of these foods, and the reasons for their food choices.

## 2. Methods

### 2.1. Study Design

This study was part of a larger mixed-methods study employing an explanatory sequential design, which also explored the extent of food marketing present in the secondary school environment. The specific elements of the study reported in this paper apply qualitative participatory research methods including workshops, the gathering of photographs via a photovoice activity, and focus group discussions (FGDs). Qualitative participatory research engages stakeholders, community members or people affected by an issue in collaboration with researchers throughout the research process to co-create knowledge in order to promote action or change [30]. Workshops were conducted among secondary school learners to gauge their knowledge and understanding of healthy and unhealthy foods. A subset of workshop participants was engaged in subsequent photovoice activity and FGDs to gather information about their SFE and further understand their perspective on the healthfulness of foods and their perceptions on marketing in their SFE. In this study the SFE was defined as the route of travel from home to school and the surrounding areas 500 metres from the school boundary and within the school compound. The photovoice activities followed the procedure outlined by Wang & Burris [28]. Photovoice and FGDs were conducted among adolescent high school learners aged between 14 and 18 years.

### 2.2. Study Site

Data were collected in two public schools in two provinces of South Africa: one school in Pretoria, Gauteng, as an urban metropolitan area and the other in Mthatha, Eastern Cape, as an urban non-metropolitan area. Both high schools are quintile five schools. South African schools are ranked in quintiles from 1 to 5 based on the socioeconomic status (SES) of the area. Quintile 1 represents the poorest schools receiving greater government support and serving communities with the lowest household income, and quintile 5 represents the least poor schools. Quintiles 1, 2 and 3 are generally declared as no-fee schools while quintiles 4 and 5 are typically fee-paying schools. Quintile 5 schools often have tuck shops with a variety of options available, and the learners are assumed to have some expendable income.

### 2.3. Sampling

**Figure 1:**
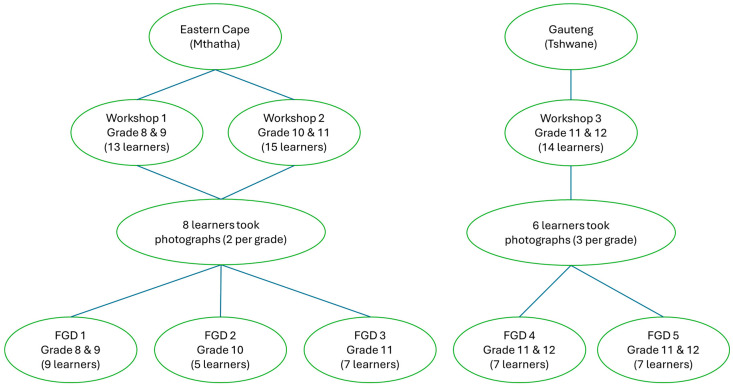
Participants in the data collection process.

The two public high schools were conveniently sampled. Adolescent learners from these public schools were purposively sampled, and thus formed a convenient sample for 3 workshops and 5 FGDs. The principals and key teachers at both schools were engaged to ensure the adequate recruitment of participants. A.K. conducted an information session on the purpose of the study for all learners from Grades 8 to 11 at the Eastern Cape school, and for all learners in Grades 11 and 12 at the Gauteng school. A registration form was given to the school administration office where interested participants could sign up to take part in the study and collect parental/guardian consent forms. Learners were included on a first-come-first-served basis with signed parental/guardian consent forms. Learners living in different suburbs and travelling on different routes to reach school were included. A total of 42 learners from both schools took part in the study voluntarily and without compensation. Thirteen to 15 learners took part in each of the 3 workshops, and between 4 and 9 learners took part in each FGD. Two learners per grade who participated in the workshops took photographs on their personal smart phones during their commute to and from home and school to capture all F&B marketing they noticed in their SFE for the photovoice study, and this material informed the FGDs. Learners for the FGDs were selected from the age groups 14–16 years for grades 8 and 9, and 16–18 years for grades 10 to 12. None of the participants withdrew from the study. Participant allocation and demographic details are provided in Figure 1 and Table 1, respectively.

### 2.4. Data Collection

Data were collected between September 2023 and May 2024 following approval from the Department of Basic Education in both provinces and permission from the school principals. The workshops, photovoice and FGDs were conducted separately for younger (Grade 8–9) and older (Grade 10–11 or 11–12) groups of students to facilitate free expression of opinions without any intimidation. Prior to data collection, pilot exercises were carried out to familiarise learners with the process, aligning it with the photovoice prompt and test photo quality. Due to safety concerns, learners only took photos where they felt comfortable and safe. Where safety was a concern, they returned to take photos accompanied by a parent or guardian.

## 3. Workshops

Initially, only 2 workshops were planned. One workshop was held with Grade 11 and 12 students in the school based in Tshwane, Gauteng. Due to the enthusiasm in the school in Mthatha, Eastern Cape, one workshop with Grades 10 and 11 learners was held and another workshop with Grade 8 and 9 learners was added. The workshop participants were asked to reflect on the following prompts: (i) would these F&Bs be classified as healthy or unhealthy and why; (ii) where is food bought by learners before, during and after school; and (iii) what are the reasons why learners choose the foods they consume? To initiate the discussion for the first prompt, photographs of 19 mixed food items, some commonly available and consumed, others considered luxury items (cherries) were shown to workshop participants, including an array of fruits, vegetables, cooked takeout food and packaged F&Bs.

### 3.1. Photo Gathering

The photovoice activity was conducted over 5 days and included 1 day of training, 1 day of photo taking practice, 2 days of actual photo taking data collection and 1 day of photo selection for discussion in the FGDs. Training included: (i) parental consent and learner assent processes; (ii) the photovoice methodology; and (iii) the ethics of photography, including no faces or aspects that can identify any individual who has not consented to it through the on-the-spot consent process. Where people were photographed, their faces were blurred out. Fourteen learners, 2 participants per grade in the Eastern Cape (Grade 8–11) and 3 participants per grade in Gauteng (Grade 11–12), took photographs on their personal smart phones during their commute to and from home and school to capture all F&B marketing they noticed in their SFE, and this material informed the FGDs.

### 3.2. Focus Group Discussions

Five FGDs with 4 to 9 participants each were conducted; 3 FGDs in the Eastern Cape and 2 in Gauteng. The focus group size was guided by research indicating that between four to six focus groups would reach at least 90% saturation in qualitative research with non-probabilistic samples [31,32]. In the Eastern Cape school, 1 FGD was conducted among Grades 8 and 9 combined, 1 with Grade 10s only and 1 with Grade 11s only. In Gauteng, 2 FGDs were conducted among Grades 11 and 12 combined. Participants viewed the 10 photographs selected from the photovoice exercise to reflect on their SFE and the marketing to which they are exposed. The following prompts guided the discussion: (i) what do you see in these photographs and how does it make you feel, and (ii) is food marketing a concern and how should it be addressed? Similar or same foods that were shown to learners in the workshop were seen in the photographs taken for the photovoice activity and this elicited further in-depth discussions on the foods learners purchase and consume, and the drivers of their choices.

The workshops and FGDs were digitally recorded using an audio recorder and field notes taken during the data collection process. Each session lasted between 45 and 65 min. All sessions were conducted in classrooms on the school premises after school. Workshops and FGDs were conducted until data saturation was reached.

### 3.3. Data Analysis

The workshops and FGDs were transcribed verbatim in English by a professional transcriber and then reviewed by A.K. for accuracy. The transcripts were coded and analysed in Atlas.ti version 25. To analyse the qualitative data, an inductive thematic analysis was conducted following the six-phase process outlined by Braun and Clarke [33] in order to develop the codebook and subsequent coding and analysis. This included identifying topics, ideas and patterns of meaning within the data. The first author (A.K.) read and coded 25% of the transcripts to develop the initial codebook. These 25% of transcripts were then double-coded by an independent researcher for standardisation purposes and differences were discussed and reviewed until agreement was reached. The codebook was then applied to all transcripts and expanded, and data were recorded to maintain rigour. Throughout this process new themes and subthemes were developed from the data.

## 4. Results

In total, 42 adolescents (14–18 years) participated in the workshops, photovoice and FGDs. Adolescents’ perceptions of healthy and unhealthy foods are discussed in two parts: (1) identifying foods, their perceived healthfulness and the frequency of their consumption, and (2) anecdotes on themes of learners’ perceptions of the factors influencing their food choices.

### 4.1. Identifying Foods, Their Perceived Healthfulness, and the Frequency of Their Consumption

Nineteen mixed types of foods were shown to learners as printed images, including fruits, vegetables, cooked takeout food and unbranded/generic packaged foods. Learners indicated whether they considered each of these foods as healthy, unhealthy or both (a combination of healthy and unhealthy), whether they recognised each of the food items and could identify them correctly, and how often they consume these foods (Table 2). The adolescents considered four of the food items to be healthy, eight to be unhealthy and seven to be a combination of healthy and unhealthy. All food items except Morogo, a traditional leafy vegetable, were recognised and correctly identified. Morogo was confused with spinach.

All the packaged foods (n = 8) and takeout foods (n = 5), except pizza (Debonairs) and ice cream (McDonald’s McFlurry), carried no known branding (generic packaging) and yet the breakfast cereals, fruit juice, cool drink (soda), and chips (crisps) were recognised and identified by popular brand names. The generic packet of chips was referred to as Lays and the cool drink as Coke.

Of the four foods that were identified as healthy, only one (apples) was eaten often. Of the eight foods identified as unhealthy, only two (granola bar and flavoured yoghurt) were not eaten often. Muesli and fruit juice were considered both healthy and unhealthy, but not often consumed.

A deeper insight into learners’ understanding of the healthfulness of these 19 foods and the context of this understanding is evident in the quotes provided in Table S1 (Supplementary Material).

### 4.2. Themes of Learners’ Perceptions of the Drivers Influencing Their Food Choices

The drivers and barriers influencing the adolescent learners’ food choices are discussed under the individual, socio-cultural and physical environment categories pertaining to their food choices.

### 4.3. Individual-Level Drivers of Adolescent Learners’ Food Choices

#### 4.3.1. Sensory Appeal, Comfort Food and Hunger

Learners often identified taste as a primary determinant of their selection of food. This was compounded by the alluring smell of certain takeout foods available in their SFE. There was a preference for takeout or store-bought packaged UPFs based on taste over home-cooked meals, especially vegetables. They reported experiencing cravings and a strong inclination towards consuming unhealthy foods as comfort foods. The appearance (sight appeal) and presentation of foods were mentioned as an influencing factor, but mainly due to their associations with taste or comfort. Hunger and satiety were mentioned as the major drivers of food choice in the SFE, and learners alluded to preferring unhealthy foods such as amagwinya/vetkoek (deep-fried dough bread) or kota (a hollowed out quarter loaf of bread often filled with fries and sauce) over healthier options such as fruits and vegetables as the unhealthy options were more filling.

*“And you’re smelling it [KFC], like yoh, mama, let me turn quickly.” (Gauteng Grade 11/12 learner)*

*“Well, I use ice cream when my life is falling apart.” (Eastern Cape Grade 11 learner.) “It’s [Kota] also very comforting. It’s filling and it feels good once you eat it. It kind of releases a hormone that makes you feel good after eating it. (Eastern Cape Grade 8/9 learner.)*

*“They sell it at the tuck shop, it’s easy to get, and on top of that, the size, the portion, it’s like, you look at it and you’re like, okay, that’s going to fill me, let me get it, and on top of that, it tastes good, so I usually get that.” (Eastern Cape Grade 8/9 learner.)*

*“No. I don’t like it [Morogo—traditional leafy vegetable]. It’s in the village. It’s mostly made there.” (Eastern Cape Grade 11 learner.)*

#### 4.3.2. Nutrition and Food Processing Knowledge

Learners referred to food processing and the addition of preservatives and additives in reference to UPFs such as packaged foods and fast foods. Regardless, the influence of taste overpowered their food choice. They were generally perceived as having some nutrition knowledge on the presence of vitamins and minerals in healthy food options and the positive health outcomes of consuming healthy foods; however, this was only a concern once a health issue persists. A discrepancy appears to exist between learners’ theoretical nutrition knowledge and their actual food choices.

*“It’s [flavoured yoghurt] less healthy because it has more additives and flavourings and colourants and all that.” (Gauteng Grade 11/12 learner.)*

*“The additives which are added into it [cool drink/soda], the flavouring additives, the colour additives, the artificial sugar, everything which is added to make it sweet.” (Eastern Cape Grade 8/9 learner.)*

*“Because for it to stay fresh and have a long shelf life, there has to be some type of process. Preservatives, yeah, added into the drinks.” (Eastern Cape Grade 8/9 learner.)*

*“Spinach is not nice, madam. I just eat it because I have to and I was once sick because I had a lack of iron.” (Eastern Cape Grade 8/9 learner.)*

### 4.4. Convenience and Time Constraints

Ordering UPFs in the form of takeout or fast foods and eating packaged snacks and beverages were noted as convenient, less time consuming and easier to do “on-the-go”, in comparison to home cooked meals. Most learners preferred not to cook meals themselves, although mothers mostly prepared meals, and those who were keen on cooking were dissuaded by the cleanup involved. Learners involved in washing dishes and cleaning up at home were frustrated by the post-cooking mess created by a parent. Learners also alluded to being “lazy” to pack lunchboxes to consume at school. Learners found it inconvenient to take fresh fruits and vegetables with them to school to consume.

*“I don’t have time in the morning to make a lunchbox. Okay, let’s say I have time in the evening, why don’t I make it in the evening? I just want to sleep. Then, okay, if I do carry a lunchbox, madam, it barely makes it to school, I finish it in the morning, on my way to school.” (Eastern Cape Grade 11 learner.)*

*“I actually thought of doing that on Friday. Just like buying … I actually thought of doing that during the weekend. Of just buying like a pack of oranges and then just keep it with me at the hostel. But then I had one problem. One, how would I get it there? And also, people would be staring at me weirdly.” (Eastern Cape Grade 8/9 learner.)*

*“But it’s so annoying especially because I’m the one who washes the dishes and is like 60 pots, for what? You cooked two things. It doesn’t make sense to me.” (Gauteng Grade 11/12 learner.)*

*“No, I love cooking, but I think it’s a scam. Because you cook for four hours, right? You eat for 10 minutes. There are dishes that you have to do. I’m sorry.” (Gauteng Grade 11/12 learner.)*

## 5. Sociocultural Drivers of Adolescent Learners’ Food Choices

### 5.1. Parental Influence and Familiarity with Foods

Several learners voiced a lack of choice in foods consumed at home, they eat what a parent has prepared or purchased, implying that parents’ knowledge and choice of healthy or unhealthy foods impact adolescent children’s food consumption. Traditional foods were only consumed if a parent or grandparent consumed them or when visiting the village.

*“At home we have a timetable, madam, on what we eat every day. We don’t eat rice. In our plate we only have potatoes, veggies and meat. We don’t have rice because we can’t have rice and potatoes on the same plate. It’s too much starch. Well my mom believes it’s too much starch. So we don’t. We don’t eat … Pap. We don’t eat … samp. Yeah, we only eat white samp but only on Saturdays. Because on Monday we have the thing that we only have veggies and meat. Then Tuesday something else.” (Eastern Cape Grade 11 learner.)*

*It [cherries] is affordable, but it depends on what our parents know.” (Gauteng Grade 11/12 learner.)*

*“Because my mom likes it [pizza]. And my brother. And so we buy it.” (Eastern Cape Grade 11 learner.)*

### 5.2. Social Status, Aspirational Food and Setting

In the school environment, learners were conscious of their peer’s food choices and careful not to appear “uncool” or “poor” preferring UPFs or branded fast foods that were considered aspirational. Although foods such as offal were liked in taste, they would not be eaten in the suburbs or at school for fear of seeming poor or backward.

*“The reason is kind of like for status. I mean, you could get a regular cone which tastes just as good, but because it’s a little bit more hyped up, you feel … You can even get ice cream for R5,00 that tastes just as good as McFlurry, but then when you have McFlurry in your hand, walking around, it’s like, wow, she has money.” (Eastern Cape Grade 8/9 learner.)*

*“And especially now with social media, everyone wants to share that, okay, I’m eating this platter with this and that and that. No one’s going to share, I’m eating a salad, we’re going to go, okay, and? You understand?” (Gauteng Grade 11/12 learner.)*

*“But this food [barbecued offal] is so ghetto, people are eating at McDonald’s, people are eating more modern food. Why would I buy this here now?” (Eastern Cape Grade 8/9 learner.)*

*“Right product [offal], wrong place.” (Eastern Cape Grade 8/9 learner.)*

## 6. Physical and Economic Environment Drivers of Adolescent Learners’ Food Choices

### 6.1. Physical Access

Learners perceived unhealthy UPFs, fast foods and beverages as widely available and accessible in their SFE and sold by both formal shops (supermarkets and franchises) and informal vendors (stalls along the street). Franchised fast foods were regarded as common in the SFE and their neighbourhoods but unavailable in the villages. Local foods such as malopies (local puffed chips), barbecued offal and other non-franchised fast foods were accessible to many learners in Gauteng travelling through town to reach school. There was no tuck shop at the school in Gauteng, and the Eastern Cape school tuck shop was only open during break time (recess) and stocked limited options mainly consisting of amagwinya/vetkoek, hotdogs, packaged ultra-processed snacks and energy drinks. Learners often had long days at school due to sport activities or extra classes and relied on the predominantly unhealthy formal and informal vendors in their SFE for meals and snacks, and fast foods were consumed as school-approved and -purchased meals on school trips.

*“In my village most of the time there is no pizzas and burgers, it’s kotas, chips, cold drinks. So it makes me feel at home.” (Gauteng Grade 11/12 learner.)*

*“Any school trips, you get KFC. It’s what they usually give for lunch.” (Eastern Cape Grade 10 learner.)*

*“For me, a lot of, not really big brands, a lot of informal stores like tents, caravans, selling junk foods, chips, crisps, sweets, that’s what I’m surrounded by.” (Eastern Cape Grade 11 learner.)*

*“Hungry Lion, it’s very popular. Right behind us [at school]” (Gauteng Grade 11/12 learner.)*

*“On my way home, I see four KFCs.” (Eastern Cape Grade 10 learner.)*

### 6.2. Economic Access

Affordability and value for money were strong influences on adolescent learners’ food purchases and consumption. The learners’ narratives indicated that healthy food options such as fruit and salads were more expensive and less filling while unhealthy fast-food options were cheaper and filling. Adolescents alluded to saving their pocket money to purchase UPFs as parents will purchase the healthier foods made available at home.

*“I’m going to buy a vetkoek [deep fried dough bread] and then I’m going to get a drink, maybe like R14. Most it would be R16. At the tuck shop I’m only going to get a hot dog for R14 rand and maybe sweets for R2. That’s all. I won’t even make it to the first lesson after break with a full stomach.” (Eastern Cape Grade 11 learner.)*

*For example, that meal we got at Science Day, it’s quite cheap because it comes with a burger, a piece of chicken, two Zinger wings, chips and a cool drink. I think it’s under R80. (Eastern Cape Grade 10 learner.)*

*“I don’t usually buy myself bananas because I know my parents at home usually have bananas, so knowing that they could buy bananas for me to buy [at school], me buying them for myself is like useless in a way.” (Eastern Cape Grade 8/9 learner.)*

*It [spending money] depends on the time of the month. For me, especially, my pocket money is to go buy a pizza.” (Gauteng Grade 11/12 learner.)*

### 6.3. Hygiene and Food Safety

A majority of the participants alluded to the hygiene and safety of the informal street vendors being questionable. Participants avoided purchasing fruits and vegetables from informal vendors sighting concerns over the cleanliness of the water and space the produce was grown or stored in, and the personal hygiene of the seller. Food items and fresh produce in the stalls were uncovered or placed directly on the ground and exposed to the elements, resulting in them being deemed unhygienic. For this reason, learners indicated a preference to purchase bananas, and other fruits and snacks, from a nearby retail chain store or a convenience store at the petrol station rather than from the informal vendor.

*It’s [vetkoek and chips vendor] so unhygienic. Also, the way he wears, like the way he dresses. And the apron, yoh [dirty]. (Gauteng Grade 11/12 learner.)*

*You don’t know if the person was from the toilet. Like there’s a bathroom, for service, then there’s toilet paper, you pay R2 to get in the bathroom and then there is chips, they are also selling chips. (Gauteng Grade 11/12 learner.)*

*“Like when they give it [barbecued gizzards] to you, like they give it in the newspaper, and it’s also unsafe because like the ink. (Gauteng Grade 11/12 learner.)*

*“Because you know, stuff sold [by the informal vendor] in this condition could be contaminated in a way. They could easily like, you know, put unknown substances into these fruits.” (Eastern Cape Grade 10 learner.)*

### 6.4. Proposed Solutions to Provide Healthier Choices

Regulating or banning the sale of unhealthy foods around school was the most common recommendation from learners, while providing access to affordable healthy foods in the SFE. Learners urged that tuck shops sell healthy cooked meals which are hygienically prepared, appetising to look at and tasty, instead of selling unhealthy foods. Several learners stated the need for teachers to adopt healthy food purchase and consumption behaviours that they could emulate. However, some learners were of the view that fast food and packaged foods have become part of their culture and would be difficult to eliminate. Others were concerned about the job losses that would occur should unhealthy food retailers and franchises be shut down.

Learners indicated that they were exposed to extensive, often contradictory, information about food on social media, and found it confusing and proposed nutrition education in schools that is relevant to the times and their context, including talks on fad diets and the harms of unhealthy foods.

## 7. Discussion

This study explored adolescent learners’ perceptions and understanding of healthy and unhealthy foods and the drivers of their food choices in their SFE in South Africa. The findings suggest the extensive popularity of and preference for UPFs, such as fast-food meals and packaged snacks and beverages, compared to healthier home-made meals. Taste preference is a substantial driver of their food choices together with affordability, availability and convenience. Branded franchise foods were preferred to their non-branded counterparts due to their perceived positive association with social status. Learners opted for foods that were satiating and considered to be value for money regardless of their negative health impacts. This study further exposes a contrast in adolescent learner’s nutrition knowledge when compared to their preferences in food purchases and consumption. Learners are generally aware of the high amounts of sugar, fat and salt in the unhealthy F&Bs they consume, and the negative health outcomes of consuming them; however, they show a greater preference for these F&Bs.

The disparity between adolescents’ nutritional knowledge and their preferences is driven by the perception that UPFs, especially branded foods, represent urban sophistication, and elevated social status among the youth, whereas healthy foods are viewed as less favourable, unhygienic or in an inferior light, aligning with evidence reported from Kenya [34]. Learners reported the lack of visual appeal in the presentation of fruits and vegetables by informal vendors as hygiene issues. Learners reported a reluctance to prepare meals at home, attributing this to feelings of laziness or to the perception that meal preparation is a responsibility typically associated with the mother, as previously reported by Erzse et al. [35]. A mother’s education and employment status has been shown to improve dietary diversity in adolescents, especially girls [36]. The adolescents were of the view that they needed time to relax and unwind by watching TV and scrolling on social media rather than preparing meals or packing lunchboxes. Practical barriers, such as lack of cooking time, cooking facilities and preference for tasty foods, inhibit actual cooking behaviour even when exposed to healthy recipes on social media [37]. Studies show that Westernised diets, consisting of mainly UPFs such as packaged F&Bs and fast food, are often consumed by young people [38]. In LMICs, children and adolescents frequently serve as early adopters of emerging F&B products [38], further highlighting the risks associated with the proliferation of fast food franchises and access to predominantly unhealthy F&Bs in the SFE, as shown in this study.

At the individual level, sensory appeal (particularly taste), hunger and convenience were the major drivers of adolescent food choices, echoing earlier evidence [34,39,40]. UPFs are intentionally engineered to have a high sensory appeal, enhanced palatability and require little to no preparation [41], rendering them convenient. A recent South African study found that 76% of packaged foods sold in the country are UPFs [42]. Learners perceived unhealthier foods to be more filling than healthier options, and this was compounded by their cost. The higher cost per gram of healthy food compared to unhealthy alternatives reduces its desirability, as it tends to be less satiating relative to its price. The strong preference for unhealthy convenient F&Bs high in nutrients of concern over healthier food options is concerning given the growing body of evidence linking unhealthy food consumption among adolescents to their poor diet quality and, in turn, to poor health outcomes such as overweight, obesity, micronutrient deficiencies [43] and cardiovascular diseases [44,45].

From a sociocultural perspective, there is a need to destigmatize nutritious traditional foods such as offal and indigenous fruits and vegetables in order to reinvent them as socially desirable and trendy. Indigenous African fruits and vegetables are nutrient-dense, sustainable and can contribute towards an affordable food supply [46]. There is a need to educate adolescents in urban settings and provide them with hands-on assistance in preparing nutritious meals that are socially desirable, affordable and convenient. Social media could be a promising medium through which to engage with adolescents and deliver educational content and healthy recipes to improve nutrition [47], provided other barriers are addressed.

Changing adolescents’ food preferences and eating behaviours will remain a challenge if their existing unhealthy SFE persists. The food environment around schools in areas of lower socioeconomic status was found to be less healthy regardless of the level of urbanisation [48,49,50]. This study found that affordable healthy meals were scarce in the SFE, while unhealthy UPFs such as fast food and packaged F&Bs were rampant and preferred, a similar finding to other studies in South African, Kenyan and Zambian SFEs [17,51,52]. South African SFEs are changing drastically into an unhealthy food environment, and multinational food companies hold most of the market share [53], infiltrating and influencing what is sold even at mini-supermarkets and tuck shops in schools. It is imperative that SFEs in South African rural areas is protected from the expansion of multinational food companies and their unhealthy products while reversing the negative shift in urban SFEs. Protecting SFEs may require a ban on the sale and marketing of unhealthy fast foods and other UPFs in SFEs across the country, while increasing the availability and accessibility, and the promotion, of healthy nutritious food and clean water to learners. Dietary patterns are dynamic and eating behaviours can change over time, especially during adolescence and early adulthood [54], making the SFE a critical space to change the food supply to eventually alter adolescents’ food preferences. Adolescents are subjected to immense unhealthy food marketing in and around schools, on TV and on social media triggering their purchase of these unhealthy options [55]. Furthermore, fiscal measures to increase taxes on unhealthy UPFs and subsidise healthy foods are required. The South African draft regulation R3337 stipulates carrying front-of-pack warning labels on foods high in sugar, fat and salt and containing any non-nutritive sweeteners, and these foods may not be marketed to children [56]. The adoption of this draft regulation would protect children in the places where they live, learn and play. Learners mentioned being influenced by potentially harmful food trends and challenges on social media and at the same time motivated to try out foods eaten by sporting role models they follow on social media. R3337 should be further enhanced to regulate unhealthy fast food in SFEs and learners’ exposure on social media.

## 8. Conclusions

In general, this study highlights adolescent learners’ perceptions of healthy and unhealthy foods and the drivers of their food choices. High school learners in both metropolitan and non-metropolitan urban areas in South Africa are seriously exposed to unhealthy foods high in critical nutrients of concern to limit. Healthy food choices are unavailable, inaccessible and unaffordable to them in their SFEs. Regardless of their theoretical knowledge of nutrition or healthy foods, they are influenced by palatability, satiety and value for money. Social status and appearing rich further fuelled the choice of branded franchise foods. Learners are influenced by visual appeal, taste and the aspirational appeal of F&Bs. The South African SFE requires multisectoral interventions to promote healthy food choices among adolescents and address the perceptions and drivers of their food choices. Mandatory government regulations banning the sale of unhealthy foods in the SFE and subsidizing healthy food options for learners should be a priority. It is recommended that the South African draft regulation R3337 be adopted at the earliest opportunity and expanded to include fast-food and other non-packaged unhealthy foods.

Further research to understand adolescent school learners’ suggestions on solutions to providing healthy foods options in the SFE is needed.

## 9. Strengths and Limitations

The use of participatory research is a particular strength of this study, empowering adolescent school-going learners to document, reflect upon and discuss in detail their lived experiences and how they determine their food choices in their SFE. The photographs taken by the learners assisted in gaining a deeper understanding of the learners SFE and allowed the researcher to probe for in-depth information, thereby enriching the insights. A potential limitation is that photography was only conducted for 2 days and more days may have allowed a longer time for reflection. This was not possible as learners were busy with their regular school schedule and data collection was facilitated with minimal disruptions. It is possible that not all school commute routes were captured in photographs or reflected upon; however, various routes and suburbs were identified and studied based on where learners lived. The in-depth insights gained are valuable; however, due to the inherent nature of qualitative participatory research, the findings may not be generalizable to the broader population.

## Figures and Tables

**Table 1:** Demographic data of the participants.

Province	Grade	Number of Participants	Age Range (Years)	Gender		
				Male	Female	Did Not Specify
Eastern Cape	Grade 8	3	14–16	0	3	
	Grade 9	10	14–16	4	6	
	Grade 10	5	16–18	1	4	
	Grade 11	10	16–18	2	6	
Gauteng	Grade 11	4	16–18	2	2	
	Grade 12	10	16–18	4	6	

**Table 2:** The healthfulness of food items, recognition of food items, and the frequency of their consumption, as indicated by adolescent learners.

Food Item	Healthfulness of Food Item as Indicated by Learners			Recognition and Identification of the Food Item by Learners		Frequency of Consumption of Food Items as Indicated by Learners	
	Healthy	Unhealthy	Combination of Healthy and Unhealthy	Correctly Identified	Incorrectly Identified/Confusion Identifying	Eaten Often	Not Eaten Often
Bananas			x	x		x	
Spinach	x			x			x
Avocados			x	x		x	
Granola bar		x		x			x
Morogo	x				x		x
Apples	x			x		x	
Cherries	x			x			x
Kota		x		x		x	
Pie			x	x		x	
Amagwinya (Vetkoek)		x		x		x	
Pizza			x	x		x	
Ice cream		x		x		x	
Chips/crisps		x		x		x	
Fruit juice			x	x			x
Cool drink/soda		x		x		x	
Marie biscuit		x		x		x	
Breakfast cereal			x	x		x	
Muesli			x	x			x
Flavoured yoghurt		x		x			x

Note: The x serves as a tick.

## Data Availability

The data presented in this study are available on request from the corresponding author. The data are not publicly available due to participant privacy, as they contain sensitive information that could compromise the anonymity of the participants.

## Supplementary Materials

The following supporting information can be downloaded at: https://www.mdpi.com/article/10.3390/ijerph23020208/s1, Table S1: Quotes pertaining to the 19 food items discussed with adolescent high school learners.
