# Supplementary material for: Urban South African Adolescents’ Perspectives on Healthy and Unhealthy Foods and the Drivers of Their Food Choices in Their School Food Environment: A Pilot Study
Source: Int J Environ Res Public Health. 2026 Feb 7;23(2):208. doi: 10.3390/ijerph23020208 (PMC12940879; doi:10.3390/ijerph23020208)
Supplement: Supplementary file 1 [file ijerph-23-00208-s001.zip › ijerph-3958026-supplementary.pdf]

Supplementary Table S1. Quotes pertaining to the 19 food items discussed with adolescent high school learners.

Number	Foods pictures displayed to learners	Quotes
1	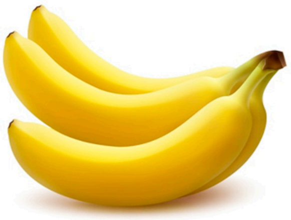 Bananas	*"My brother eats bananas every day. I think he's addicted to bananas. My mom buys it." (Gauteng Grade 12 learner)*<br><br>*"I bring fruit … two fruits every day, an apple and a banana." (Eastern Cape Grade 8/9 learner)*<br><br>*"…it's healthy, but I think you should also view how much of it you take. Especially with bananas, they are very high in potassium, and sometimes that's not very good for your body. So it is healthy because it is a fruit, but don't [eat] excessively." (Gauteng Grade 11/12 learner)*<br><br>*"When it comes to whether I would eat it and how often I would eat it, I live in a school hostel, so I don't necessarily get a choice of what I eat, because it's usually about what they give. And at the hostel, they usually give us fruit once a week, and then that's sometimes. Sometimes they don't give us fruit at all." (Eastern Cape Grade 8/9 learner)*

2	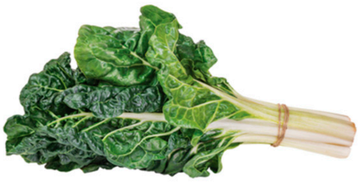<br>Spinach	*"Every single way I've tried to eat spinach, guys, is just not appetizing." (Eastern Cape Grade 8/9 learner)*<br><br>*'No, it's different taste buds. Honestly. Like we can cook it the same way. You like it, I won't. It's just the way it is.' (Gauteng Grade 11/12 learner)*<br><br>*It's one of my favourite green vegetables.*<br><br>*I only eat it when I'm at home. Since I live in the hostel, they don't usually serve it." (Eastern Cape Grade 11 learner)*<br><br>*"I just eat it because I have to and I was once sick because I had a lack of iron." (Eastern Cape Grade 8/9 learner)*<br><br>*"If you touch someone and you feel electric static, they say if you eat spinach it stops that." (Gauteng Grade 11/12 learner)*
3	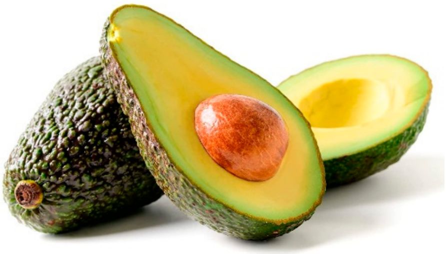<br>Avocados	*"As one person who eats a lot of avocados, I can say it is very unhealthy. Because of the fat. The amount of fat that's in it. So, maybe, no, actually no, let me say it is healthy because it is a vegetable but then again we go back to the excessiveness of eating whatever (fat)." (Gauteng Grade 11/12 learner)*

		"Because I know that avocados are high in fat, but then some people say that they're high in the good fats, and then some people say that good fats don't exist at all. So then I'm not sure." (Eastern Cape Grade 8/9 learner)<br><br>*"Oh, she doesn't like saying that [that Avocados are healthy] because she doesn't like avocados." (Eastern Cape Grade 11 learner)*
4	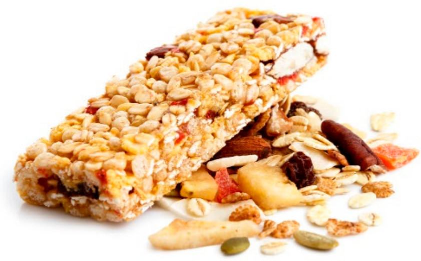<br>Granola bar	*"I don't necessarily know why, but then people usually say that it's healthy. And then when they're advertising, they'll show things like fitness and just keeping healthy and stuff." (Eastern Cape Grade 8/9 learner)*<br><br>*"It's also because the way they make the granola bars, they are processed. They have things added into them to make it more appealing. So when you taste it, you want more." (Eastern Cape Grade 8/9 learner)*<br><br>*"I say unhealthy madam because, yes, nuts, raisins, all that tastes good, but the whole reason they taste nice in that form is because of the amount of sugar it has. They even put chocolate at the bottom to make it taste better, so it's only the sugar." (Eastern Cape Grade 8/9 learner)*

5	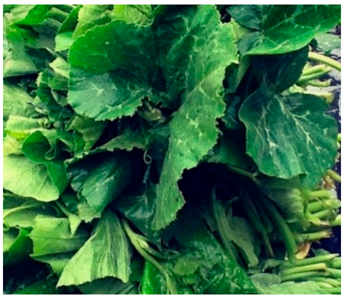<br><br>Morogo	*"It's very healthy shame, but it's not nice." (Gauteng Grade 11/12 learner)*<br><br>*"No. I don't like it. It's in the village. It's mostly made there." (Eastern Cape Grade 11 learner)*<br><br>*"Twice a week. In the week it's prepared at home, and my granny only loves it" (Eastern Cape Grade 11 learner)*<br><br>*"Maybe twice a year [consumption], madam" (Eastern Cape Grade 11 learner)*
6	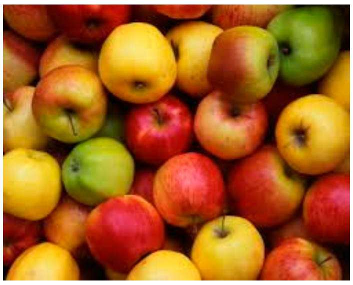<br><br>Apples	*"Very nice. Especially the greener the better." (Gauteng Grade 11/12 learner)*<br><br>*"Apples are juicy." (Eastern Cape Grade 8/9 learner)*<br><br>*I don't eat them often because they are not available [in hostel]." (Eastern Cape Grade 11 learner)*
7	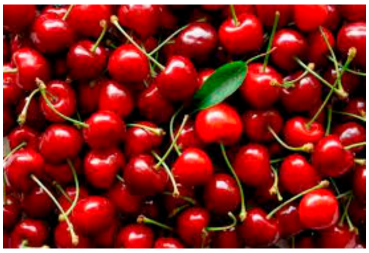<br><br>Cherries	"I never tasted a cherry in my life." *(Eastern Cape Grade 8/9 learner)*<br><br>"It [cherries] is affordable, but it depends on what our parents know." *Gauteng Grade 11/12 learner)*

		“It's freshly sourced, they’re not processed.” *(Eastern Cape Grade 8/9 learner)*<br><br>*“The natural ones are sour. And then maraschino cherries that like they put like maybe in black forest, it’s like covered in syrup or something.” (Gauteng Grade 11/12 learner)*<br><br>“Cherries are more expensive than apples, so when you have the option which is more available for you, you rather choose the one which will cost you less.” *(Eastern Cape Grade 8/9 learner)*<br><br>“Cherries are relatively smaller than apples, so I guess there'll be more healthy benefits in an apple than a cherry.” *(Eastern Cape Grade 8/9 learner)*
8	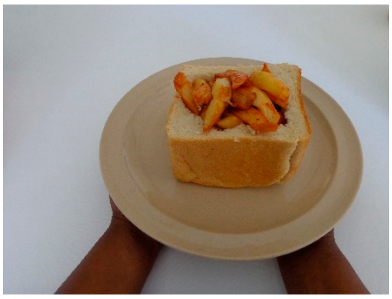<br><br>Kota<br><br>(hollowed out quarter loaf of bread often filled with fries and a sauce)	*“I also think presentation-wise, it looks very appetizing compared to other healthy foods. You see nuts, who would go for nuts when you have a whole kota?” (Eastern Cape Grade 8/9 learner)*<br><br>*“Yes, and it's cheaper than the healthier foods that are offered.” (Eastern Cape Grade 8/9 learner)*<br><br>*There's only one option, it's the kota, the chips and the coke. (Gauteng Grade 11/12 learner)*

		"It's the closest thing there, so why not … why go somewhere further away to get something healthy if it's right there?" (Eastern Cape Grade 8/9 learner)<br><br>*"I don't buy kota. I don't. I do eat it, but I don't buy it. Because, I don't know, first of all, it's not so close, and I'm always rushing somewhere, or I'm really lazy to walk." (Eastern Cape Grade 11 learner)*
9	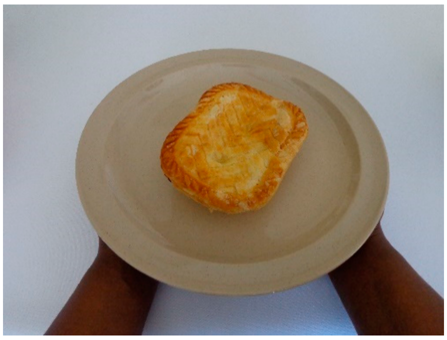<br>Pie	*"It melts my heart." (Eastern Cape Grade 8/9 learner)*<br><br>*"It depends [healthfulness]. Yeah, there's veggies [inside the pie]." (Gauteng Grade 11/12 learner)*<br><br>*"Yeah, I get those veggie pies, those are healthy." (Gauteng Grade 11/12 learner)*<br><br>*"I want to argue. That's healthy. Because the pastry, yeah, it's flour and then eggs. Okay like, they can also be unhealthy but there's also, okay, maybe it is unhealthy, but compared to other things that we've seen, it's healthy." (Eastern Cape Grade 11 learner)*

		"The vegetables. Yeah, there's veggies [inside the pie]. And then there's just one maybe like sausage …" (Gauteng Grade 11/12 learner)<br><br>*"And the meat, okay, like if steak and kidney, chicken and mushroom, there's veggies and then there's also a bit of meat."*
10	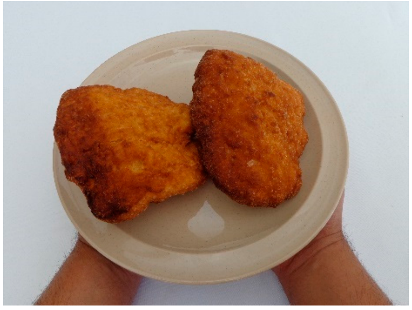<br>Amagwinya (Vetkoek)<br>(deep fried dough bread/ vetkoek)	*"That's my love." (Eastern Cape Grade 8/9 learner)*<br><br>*"It's at the gate." (Gauteng Grade 11/12 learner)*<br><br>*"Hashtag hungry and broke. Because it catches you after school." (Gauteng Grade 11/12 learner)*<br><br>*"The amount of oil it needs to cook [makes it unhealthy]." (Eastern Cape Grade 8/9 learner)*<br><br>*"They sell it at the tuck shop, it's easy to get, and on top of that, the size, the portion, it's like, you look at it and you're like, okay, that's going to fill me, let me get it, and on top of that, it tastes good, so I usually get that." (Eastern Cape Grade 8/9 learner)*<br><br>*"When we look at take-out, we look at it as, okay, mom's not cooking, so we're going to go to*

		McD's and then buy supper. So, we have McD's for supper. That's how we look at take-outs. So, it's [Amagwinya and Kota] just available comfort food." (Eastern Cape Grade 11 learner)
11	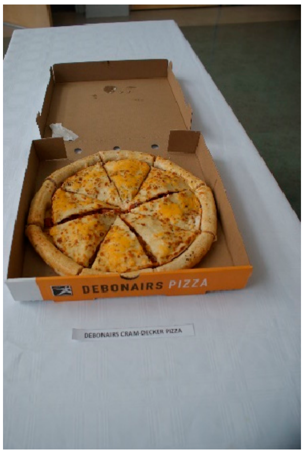<br><br>Pizza	*"Since like Debonairs pizza is like a very well established brand, the name itself also promise you to give you quality." (Eastern Cape Grade 8/9 learner)*<br><br>*"Because my mom likes it. And my brother. And so, we buy it." (Eastern Cape Grade 11 learner)*<br><br>*"I think it's like the brand which Debonairs has built up from like since it was created. Since people know Debonairs pizza as, yeah, the good pizza. Unlike when you compare to let's say, Roman's pizza or Panarotti's." (Eastern Cape Grade 8/9 learner)*<br><br>*It's delicious. Not only, you know, madam, it's Debonairs pizza." (Eastern Cape Grade 8/9 learner)*<br><br>*"Some of us prefer to eat veggie pizza. [Alluding to its healthy property]" (Gauteng Grade 11/12 learner)*
12		*"I eat ice cream every day. [Not necessarily McFlurry]" (Eastern Cape Grade 11 learner)*

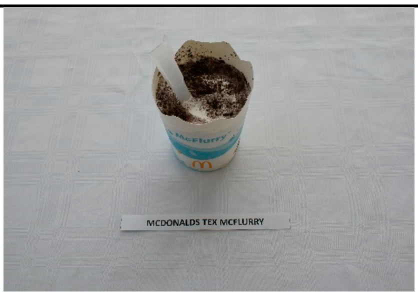

McFlurry icecream

“For me when it comes to McFlurry, I feel like the only reason why I would necessarily choose that ice cream in particular over other ice creams, is because of the name.” (Eastern Cape Grade 8/9 learner)

“I'd say ice cream is seen as like a comfort food, or any sweet treats are seen as a comfort food.” (Eastern Cape Grade 8/9 learner)

13

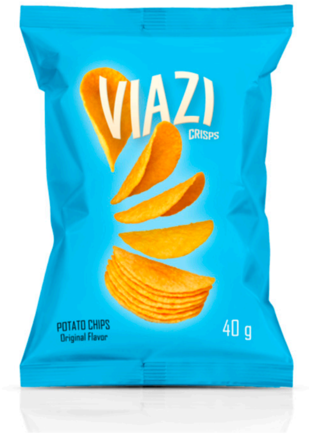

Packaged chips/ crisps

“Lays to be specific” (Gauteng Grade 11/12 learner)

“Because it's cheap. Some of us are not financially stable, so we just buy it.” (Eastern Cape Grade 11 learner)

“Yeah. Boredom [reason to consume it].” (Eastern Cape Grade 11 learner)

“Cravings [reason to consume it].” (Eastern Cape Grade 11 learner)

14

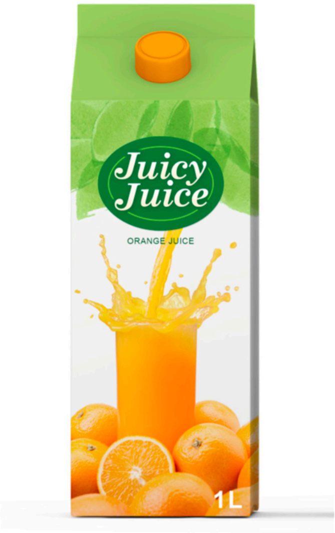

Packaged fruit juice

"I'd say it's kind of in between, I guess it kind of depends on the brand, because there are some brands which kind of offer a little bit of pureness, but then there's others that offer like 100% artificial drinks." (Eastern Cape Grade 8/9 learner)

"Some are sweetened juice." (Eastern Cape Grade 11 learner)

"Honestly, I don't know, but I know I drink a lot of water. I try to finish a bottle of 4.5 litres of water a day." (Eastern Cape Grade 11 learner)

"Do we actually like water or do we not afford the juice?" (Eastern Cape Grade 11 learner)

15

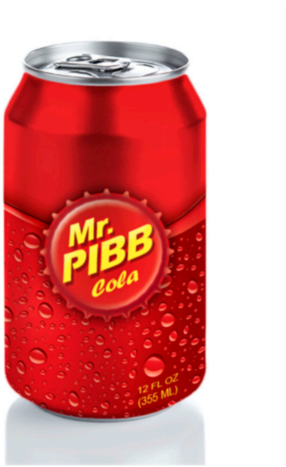

Cooldrink/ Soda

"There is too much acid and sugar." (Eastern Cape Grade 11 learner)

"Everyday [consumed]" (Gauteng Grade 11/12 learner)

"The only reason the drink itself tastes good is because of the high amount of sugar the drink itself contains." (Eastern Cape Grade 8/9 learner)

		"The acid, the taste, the aroma. Just that it's satisfying." (Gauteng Grade 11/12 learner)
16	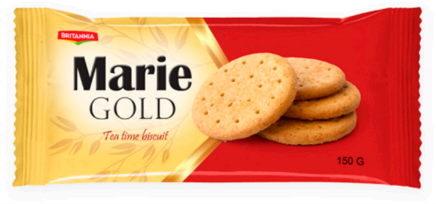<br><br>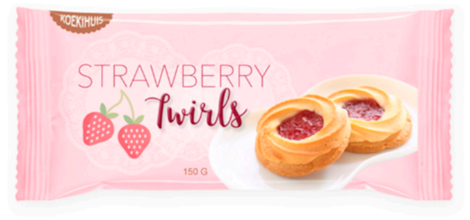<br><br>Packaged biscuits	*"[Unhealthy] because it has a lot of sugar." (Gauteng Grade 11/12 learner)*<br><br>*"Okay, like at least like four times a week [consumption]." (Eastern Cape Grade 11 learner)*<br><br>*"These biscuits are made so that they last longer and also made with ingredients so that they are much more cheaper and quicker to get and make. So they don't really care about how healthy it is, as when you yourself are making them from scratch at home." (Eastern Cape Grade 8/9 learner)*
17	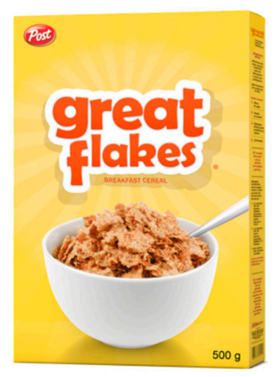<br><br>Breakfast cereal	*"Yeah, with plain yogurt and fruit. And then there are those people that eat it with sugar, and especially with like Coco Pops or something, there are people that add the additional sugar." (Gauteng Grade 11/12 learner)*<br><br>*I'm not sure, but I think it's because it is processed. It's not directly, I don't know whether they used natural products to make it or not. (Eastern Cape Grade 11 learner)*

		"Yeah, with plain yogurt and fruit. And then there are those people that eat it with sugar, and especially with like Coco Pops or something, there are people that add the additional sugar." (Gauteng Grade 11/12 learner)<br><br>*"Every day [consumption]." (Eastern Cape Grade 11 learner)*
18	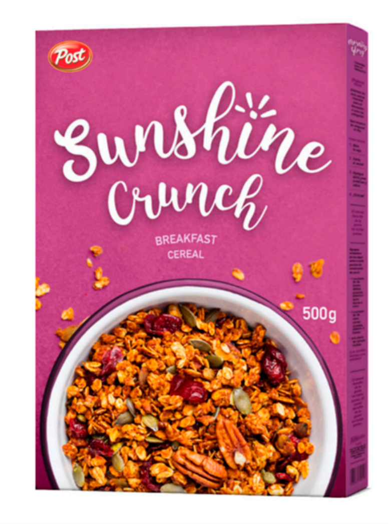<br>Muesli	*"Doesn't that have like sugar in it." (Gauteng Grade 11/12 learner)*<br><br>*"Because the muesli that I eat has actually got sugar in it. I actually read the box because I always ask myself, why does it taste so sweet regardless of what you add to it? Because I've done a comparison where I used pure oats and I added yogurt to it, and then I added yogurt to muesli, and I tasted the difference. It tastes less plain but it's more filling, it makes you more full. And then the other one tastes sweeter regardless of what you add to it." (Eastern Cape Grade 11 learner)*
19	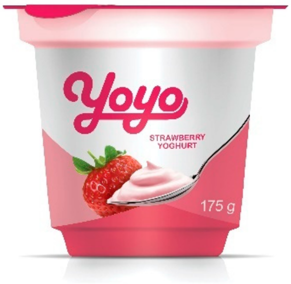<br>Flavoured yoghurt	*"I prefer plain yoghurt. It's [flavoured yoghurt] less healthy because it has more additives and flavourings and colourants and all that." (Gauteng Grade 11/12 learner)*<br><br>*"My mother say they're healthy because most of the yogurt contains a lot of minerals that are healthy for your body and your*

	brain too." (Gauteng Grade 11/12 learner)<br><br>*"I like reading packages, especially of these things. Most yogurts that have flavours like strawberry, mango, they are sweetened. It's not only the fruit that goes in, but like additional sugar also goes in to make it sweeter and some oils." (Eastern Cape Grade 11 learner)*<br><br>*"I'm saying it depends because they are ... I don't know if I should say they are healthy, the ones that are dairy free and fat free and whatever, but then it is, I don't know if it's stats or what, but they are like plain yogurt that is considered to be healthy than the actual flavoured ones." (Gauteng Grade 11/12 learner)*<br><br>*"It's less healthy because it has more additives and flavourings and colourants and all that." (Gauteng Grade 11/12 learner)*
